# Supplementary material for: Protein Interactome Analysis of the Type IX Secretion System Identifies PorW as the Missing Link between the PorK/N Ring Complex and the Sov Translocon
Source: Microbiol Spectr. 2022 Jan 12;10(1):e01602-21. doi: 10.1128/spectrum.01602-21 (PMC8754138; doi:10.1128/spectrum.01602-21)
Supplement: SUPPLEMENTAL FILE 1 — Supplemental material. Download SPECTRUM01602-21_Supp_1_seq8.pdf, PDF file, 0.1 MB [file spectrum01602-21_supp_1_seq8.pdf]

SUPPLEMENTARY MATERIAL

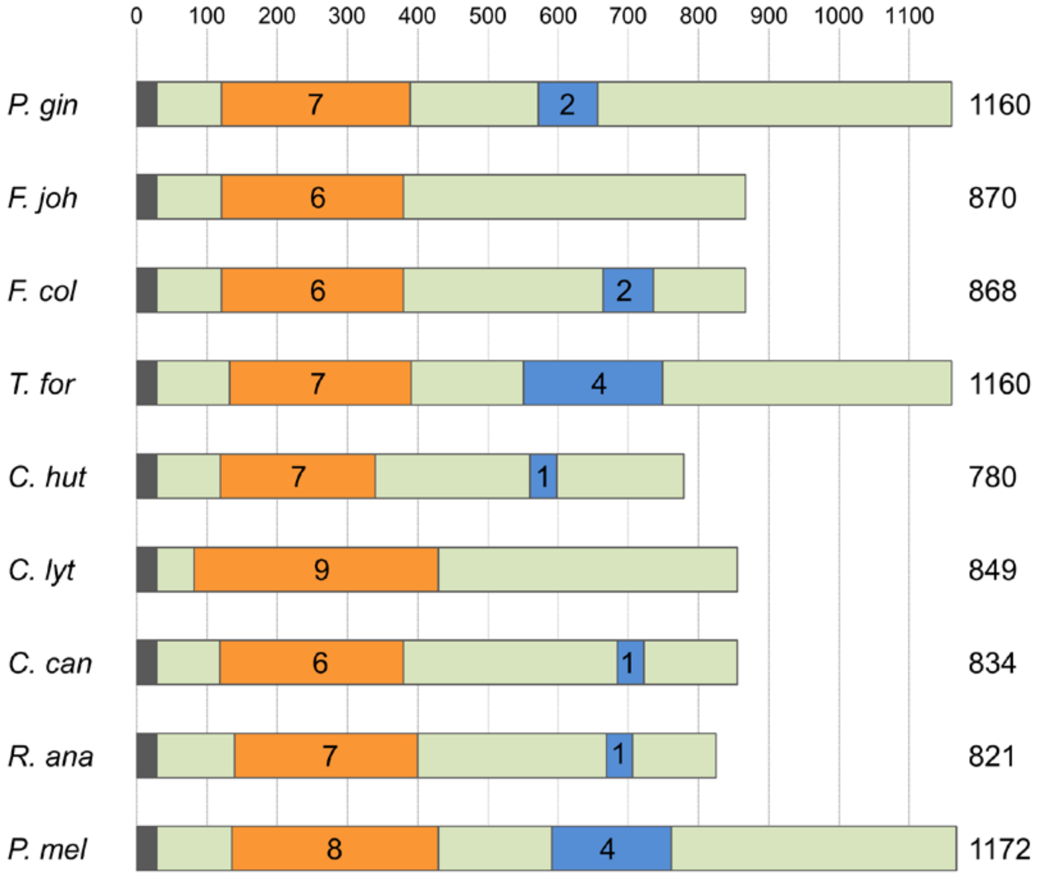

Supplementary Figure 1: TPR motifs in T9SS PorW/SprE proteins

Supplementary Table 1. Strains, Plasmids and Oligonucleotides used in this study.

Strains	Description and genotype	Source/References
Escherichia coli K12		
DH5α	F-, Δ(*argF*-*lac*)U169 *phoA supE44* Δ(*lacZ*)M15 *recA relA endA thi hsdR gyr*	Cascales Laboratory
W3110	F-, lambda-IN(*rrnD*-*rrnE*)1 *rph*-1	Cascales Laboratory
BTH101	F-, *cya99, araD139, galE15, gakK16, rpsL, hsdR, mcrAB*	(Karimova et al. 1998)
Porphyromonas gingivalis		
DSM20709	WT *Porphyromonas gingivalis* (ATCC33277/DSM20709)	DSMZ collection
ATCC 33277 ABK-	Gingipains-null *Porphyromonas gingivalis* mutant	Nakayama Laboratory
ATCC 33277 *sov*	Δ*sov Porphyromonas gingivalis*	Nakayama Laboratory
ATCC 33277 *porW*	Δ*porW Porphyromonas gingivalis*	Nakayama Laboratory
ATCC 33277 *porP*	Δ*porP phyromonas gingivalis*	Nakayama Laboratory
ATCC 33277 *porT*	Δ*porT phyromonas gingivalis*	Nakayama Laboratory
ATCC 33277 *porV*	Δ*porV Porphyromonas gingivalis*	Reynolds Laboratory
ATCC 33277 *porD*	Δ*porD Porphyromonas gingivalis*	Reynolds Laboratory

Plasmid	Description and main characteristics	Source
pASK-IBA4	Expression vector, AHT-inducible, Amp^R	IBA technology
pIBA4-$PorW_{FL}$	*P. gingivalis porW* gene cloned into pASK-IBA4, C-terminal FLAG epitope	This study
pIBA4-PorW-$D6_{FL}$	*P. gingivalis porW* D6 region (aa 1052-1161) cloned into pASK-IBA4, C-terminal FLAG epitope	This study
pBAD33	Expression vector, AraC, arabinose-inducible, Cm^R	(Guzman et al. 1995)
pBAD-$PorN_V$	*P. gingivalis porN* gene cloned into pBAD33, N-terminal VSV-G epitope	(Vincent et al. 2017)
pBAD33-ssOmpA	pBAD33, Shine-Dalgarno and OmpA signal sequence	Dukas Jurėnas, unpublished
pBADss-Sov_V	*P. gingivalis sov* gene cloned into pBAD33-ssOmpA, C-terminal VSV-G epitope	This study
pUT18C	BACTH vector, ColE1 origin, *P*lac, T18 domain of *Bordetella* adenylate cyclase, Amp^R,	(Karimova et al. 1998)
pT18-Pal	*E. coli pal* without signal sequence cloned downstream T18 into pUT18C	(Battesti and Bouveret 2006)
pT18-PorW	*P. gingivalis porW* without signal sequence cloned downstream T18 into pUT18C	This study
pT18-PorK	*P. gingivalis porK* without signal sequence cloned downstream T18 into pUT18C	(Vincent et al. 2017)
pT18-$PorL_C$	*P. gingivalis porL* cytoplasmic domain (aa 73-309) cloned downstream T18 into pUT18C	(Vincent, Durand, and Cascales 2016)
pT18-$PorM_P$	*P. gingivalis porM* periplasmic domain (aa 36-516) cloned downstream T18 into pUT18C	(Vincent et al. 2017)

pT18-PorN	*P. gingivalis porN* without signal sequence cloned downstream T18 into pUT18C	(Vincent et al., 2017)
pKT25	BACTH vector, P15A origin, *Plac*, T25 domain of *Bordetella* adenylate cyclase, Kan^R	(Karimova et al., 1998)
pT25-TolB	*E. coli tolB* without signal sequence cloned downstream T25 into pKT25	(Battesti and Bouveret, 2006)
pT25-PorW	*P. gingivalis porW* without signal sequence cloned downstream T25 into pKT25	This study
pPorW-T25	*P. gingivalis porW* without signal sequence cloned upstream T25 into pKT25	This study
pT25-PorW-TPR1	*P. gingivalis porW* TPR1 domain (aa 125-400) cloned downstream T25 into pKT25	This study
pT25-PorW-TPR2	*P. gingivalis porW* TPR2 domain (aa 589-684) cloned downstream T25 into pKT25	This study
pT25-PorW-D1	*P. gingivalis porW* D1 region (aa 1-94) cloned downstream T25 into pKT25	This study
pT25-PorW-D2	*P. gingivalis porW* D2 region (aa 95-258) cloned downstream T25 into pKT25	This study
pT25-PorW-D3	*P. gingivalis porW* D3 region (aa 259-388) cloned downstream T25 into pKT25	This study
pT25-PorW-D4	*P. gingivalis porW* D4 region (aa 389-788) cloned downstream T25 into pKT25	This study
pT25-PorW-D5	*P. gingivalis porW* D5 region (aa 789-1051) cloned downstream T25 into pKT25	This study
pT25-PorW-D6	*P. gingivalis porW* D6 region (aa 1052-1161) cloned downstream T25 into pKT25	This study
pT25-PorW-D7	*P. gingivalis porW* D7 region (aa 95-788) cloned downstream T25 into pKT25	This study
pT18-PGN1783	*P. gingivalis pgn_1783* without signal sequence cloned downstream T18 into pUT18	This study
pPGN1783-T18	*P. gingivalis pgn_1783* without signal sequence cloned upstream T18 into pUT18	This study
pT25-PGN1783	*P. gingivalis pgn_1783* without signal sequence cloned downstream T25 into pKT25	This study
pPGN1783-T25	*P. gingivalis pgn_1783* without signal sequence cloned upstream T25 into pKT25	This study

**Oligonucleotide**	**Sequence (5' to 3')**
Plasmid construction [a,b,c]	
5-IBA4-PorW	GCAGTGGCACTGGCTGGTTTCGCTACCGTACTCCTATCTGGGTGCTCCACATCGAAGAATACGGCG
3-IBA4-PorW-FL	GGTGGCTCCAGCTAGCGGCCTGCGCTTA*TTTATCATCGTCGTCTTTATAATC*TTGGGGCCCTCCGCCG
5-IBA4-D6-FL	GTTTCGCTACCGTAGCGCAGGCCGCTGCCAAGCGAGAAAAGGAGC
3-IBA4-D6	GCCTTTTTCGAACTGCGGGTGGCTCCAGCTTTA*TTTATCATCGTCGTCTTTATAATC*TTGGGGCCCTCCGCC
5-pBADss-Sov-SalI	GAAG**GTCGAC**GGCTCCGTACCTGCCC
3-pBADss-Sov-VSVG-PstI	GAAG**CTGCAG**TTA*TTTTCCTAATCTATTCATTTCAATATCTGTATA*CTGCGTCAGATTGAAACGG
5-BACTH-PorW-XbaI	GAAG**TCTAGA**TTCCACATCGAAGAATACGGCGG
3-BACTH-PorW-KpnI	GAAG**GGTACC**CCTTGGGGCCCTCCGCC
5-BACTH-TPR1-XbaI	GAAG**TCTAGA**TAAAGCCGTAGCTTTCCAGAAC
3-BACTH-TPR1-Kpn1	GAAG**GGTACC**CCATTGAGTCTGTTCAATACACCGGC

5-BACTH-TPR2-XbaI	GAAG**TCTAGA**TATGACGGAAGATGCCAAGGAAG
3-BACTH-TPR2-KpnI	GAAG**GGTACC**CCATTGCTCAGGGCCTTTGCC
3-BACTH-D1-KpnI	GAAG**GGTACC**CCACCTTGCTTACGCTCCGG
5-BACTH-D2-XbaI	GAAG**TCTAGA**TTGGCAGAGCGATCCCAAAG
3-BACTH-D2-KpnI	GAAG**GGTACC**CCCGGAGAAGCCCGCAACAC
5-BACTH-D3-XbaI	GAAG**TCTAGA**TCCTTTCGCTTTGGACTTTGCC
3-BACTH-D3-KpnI	GAAG**GGTACC**CCCAATTCATCGAGCCGGGAGG
5-BACTH-D4-XbaI	GAAG**TCTAGA**TGTCGGTCATGCCAAGGTCG
3-BACTH-D4-KpnI	GAAG**GGTACC**CCAGGCAAAGAGTCCGGCG
5-BACTH-D5-XbaI	GAAG**TCTAGA**TCCTTTTGTAAAACCCAAATCTTTCGAGC
3-BACTH-D5-KpnI	GAAG**GGTACC**CCCTTTTCGAGTGCTTTCTTTTCGTCC
5-BACTH-D6-XbaI	GAAG**TCTAGA**TGCCAAGCGAGAAAAGGAGC
T25T18C-5-PGN1783	CGGATAACAATTTCACACAGGAAACAGCTATGACCATGGCTGTAAGTCAGGAGTTGAATGCCAAGG
T18C-3-PGN1783	CCTCGCTGGCGGCTAAGCTTGGCGTAATTTTTTTGAGCGGATTCAGCTTGTTTCCTTCAG
T25C-3-PGN1783	GTTTGCGTAACCAGCCTGATGCGATTGCTGTTTTTTGAGCGGATTCAGCTTGTTTCCTTCAG
T18N-5-PGN1783	CGCCACTGCAGGGATTATAAAGATGACGATGACAAGGCTGTAAGTCAGGAGTTGAATGCCAAGG
T25N-5-PGN1783	GGCGGGCTGCAGATTATAAAGATGACGATGACAAGGCTGTAAGTCAGGAGTTGAATGCCAAGG G
T25T18N-3-PGN1783	CGAGGTCGACGGTATCGATAAGCTTGATATCGAATTCTAGTTATTTTTTGAGCGGATTCAGCTTGTTTCCTTCAG

[a] sequence annealing on target vector underlined.
[b] FLAG- or VSV-G-coding sequence italicized
[c] Restriction site in bold.

SUPPLEMENTARY REFERENCES

Battesti, A., and E. Bouveret. 2006. 'Acyl carrier protein/SpoT interaction, the switch linking SpoT-dependent stress response to fatty acid metabolism', *Mol Microbiol*, 62: 1048-63.

Guzman, L. M., D. Belin, M. J. Carson, and J. Beckwith. 1995. 'Tight regulation, modulation, and high-level expression by vectors containing the arabinose PBAD promoter', *J Bacteriol*, 177: 4121-30.

Karimova, G., J. Pidoux, A. Ullmann, and D. Ladant. 1998. 'A bacterial two-hybrid system based on a reconstituted signal transduction pathway', *Proc Natl Acad Sci U S A*, 95: 5752-6.

Vincent, M. S., M. J. Canestrari, P. Leone, J. Stathopulos, B. Ize, A. Zoued, C. Cambillau, C. Kellenberger, A. Roussel, and E. Cascales. 2017. 'Characterization of the *Porphyromonas gingivalis* Type IX Secretion Trans-envelope PorKLMNP Core Complex', *J Biol Chem*, 292: 3252-61.

Vincent, M. S., E. Durand, and E. Cascales. 2016. 'The PorX Response Regulator of the *Porphyromonas gingivalis* PorXY Two-Component System Does Not Directly Regulate the Type IX Secretion Genes but Binds the PorL Subunit', *Front Cell Infect Microbiol*, 6: 96.
